# Supplementary material for: Isolation and Characterization of Extracellular Vesicles Derived from Mango Fruits
Source: Int J Mol Sci. 2025 Nov 25;26(23):11375. doi: 10.3390/ijms262311375 (PMC12691788; doi:10.3390/ijms262311375)
Supplement: Supplementary file 1 [file ijms-26-11375-s001.zip › Figures S1 and S2.pdf]

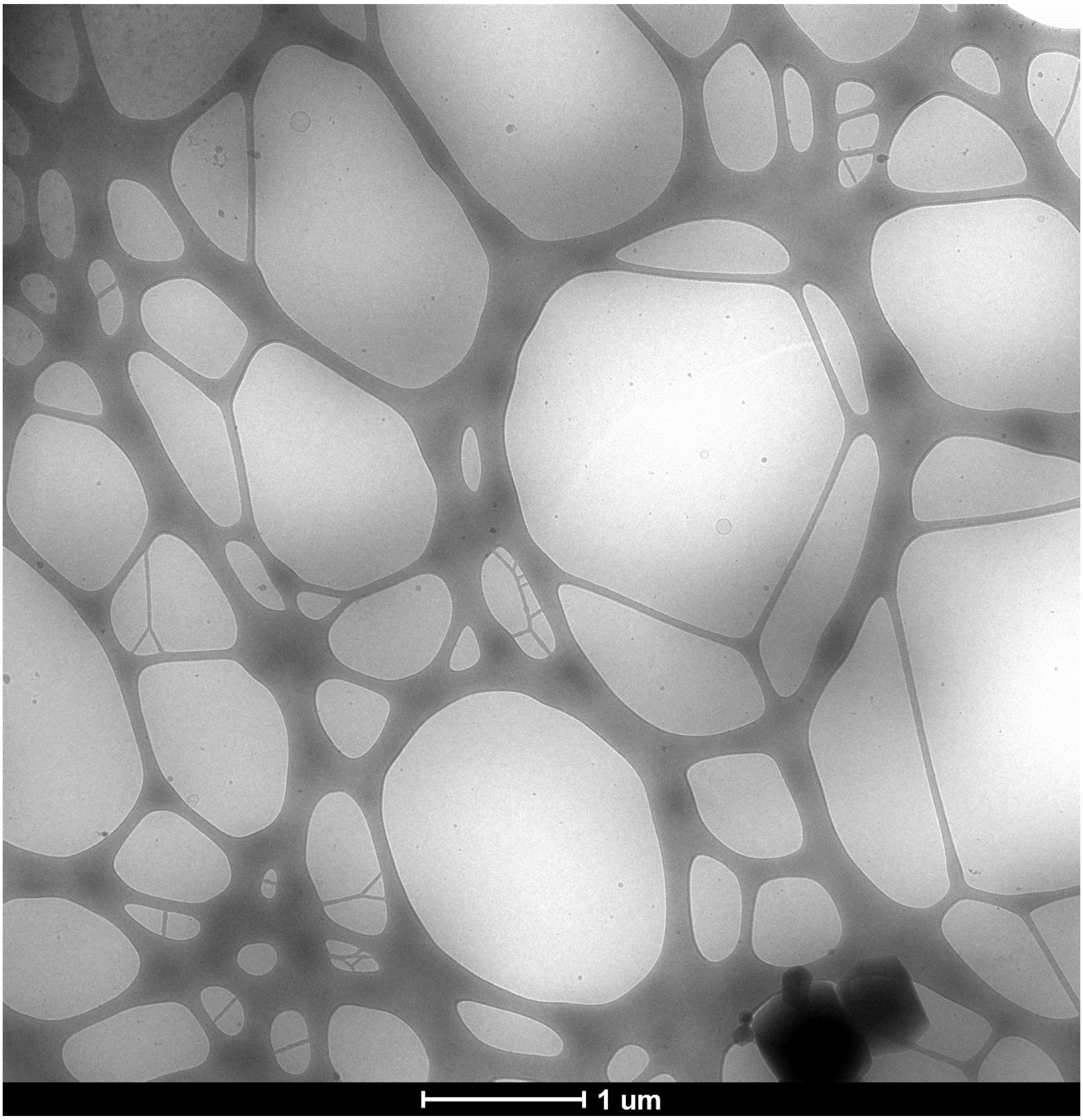

Figure S1. Zoom-out cryo-TEM image of EV isolate presented in Figure 3B.

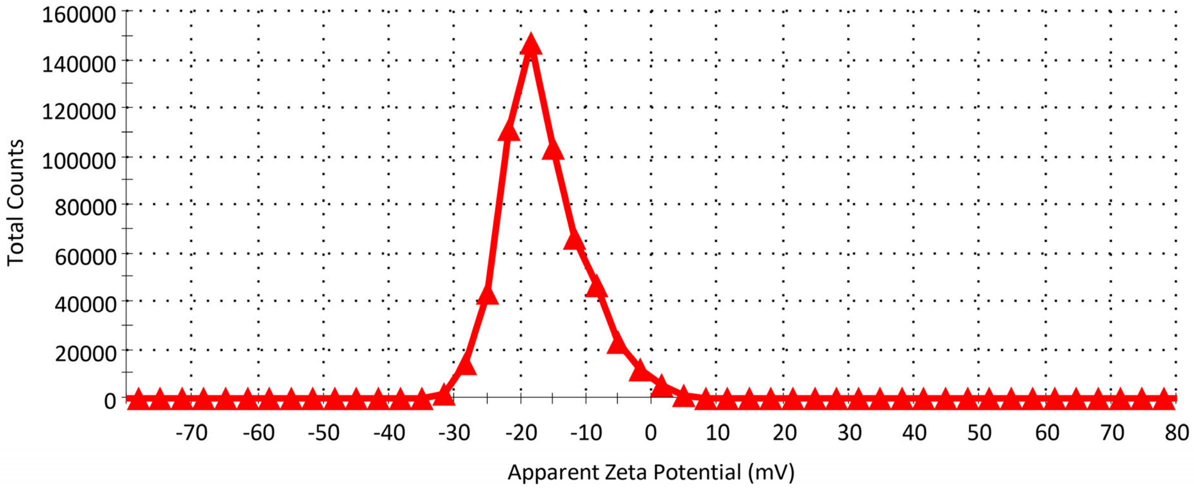

Figure S2. Zeta potential measurements of mango-derived EVs.
